# Expanding the Phenotype: A Case Report of a Novel Alanyl-tRNA Synthetase 2 (AARS2) Homozygous Mutation in a 17-Month-Old Child

**DOI:** 10.7759/cureus.86330

**Published:** 2025-06-19

**Authors:** Hadi A Helali, Samar Almuntaser

**Affiliations:** 1 Pediatric Neurology, Al Jalila Children's Speciality Hospital, Dubai, ARE

**Keywords:** alanyl-trna synthetase 2, dystonia, metabolic disease, mitochondrial disease, novel mutation

## Abstract

Early developmental delay and progressive neurological symptoms are red flags when evaluating infants in neurology clinics. Early assessment and management are essential to improve outcomes, with genetic testing being a cornerstone. Even if initial genetic results were not suggestive, revisiting the reported variant and comparing it to the newly published reports of different phenotypes helps establish the clinical diagnosis, as the authors report in this case of a 17-month-old child who presented with global developmental delay and hypotonia and was found to have Alanyl-tRNA Synthetase 2 (*AARS2*) gene mutation that was deemed as variant of uncertain significance but now is believed to be pathogenic and responsible for her evolving clinical manifestations.

## Introduction

Alanyl-tRNA Synthetase 2 (AARS2) is a nuclear-coded enzyme that is responsible for the proper formation and function of aminoacyl-tRNA synthetase, a mitochondrial enzyme that has an essential role in oxidative phosphorylation and ATP generation by the mitochondria, required for normal function of many organs in the human body [1]. Mutations in this gene leading to disease were first reported in 2011 in three infants who died because of hypertrophic cardiomyopathy, leading to heart failure [2]. Since then, more cases have been reported, with a broader range of different mutations and a varying spectrum of clinical manifestations [3]. That being said, until now, the mutation and its associated diseases are still considered an ultra-rare entity, with the reported number of patients being sixty worldwide so far [3,4]. In this article, the authors report a case of a novel homozygous mutation in the *AARS2* gene in a 17-month-old Middle Eastern girl presenting with global developmental delay, hypotonia, and who has been followed up until she is now 10 years old and has started to develop dystonia as well.

## Case presentation

The girl was 17 months old on her first presentation to the pediatric neurology department. She was born full term by normal vaginal delivery with no immediate post-natal concerns. The parents are first-degree maternal cousins from two different Middle Eastern countries, with no reported family history of developmental delay or regression in the family. The child started sitting with support at the age of 15 months and started to stand up with support at 17 months of age. She could not speak any words then, nor could she indicate her needs or understand commands. This caused concern in the parents, so they brought her to the clinic.

On her first neurological assessment, she was found to have normal growth parameters, with weight, height, and head circumference within the 50th percentiles for age. She did not have any dysmorphic features. She was observed to have occasional horizontal nystagmus, but no other abnormalities on cranial nerve examinations. She had both axial and appendicular hypotonia with brisk knee reflexes. She still had neonatal reflexes present at that time, with strong grasp reflex bilaterally.

**Figure 1:**
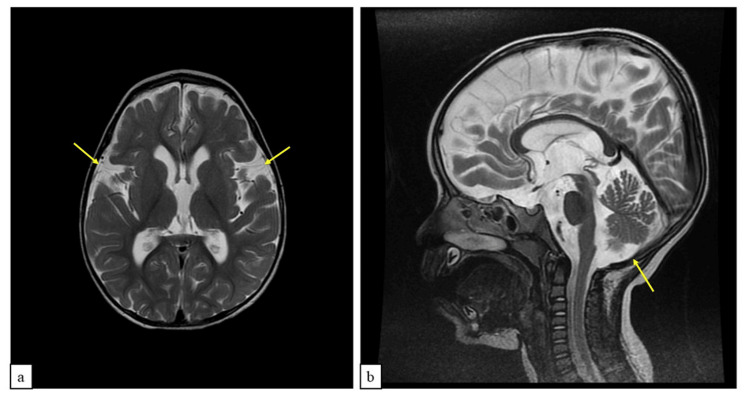
T2 MRI images of the child showing prominent cerebellar folia and brain volume reduction a. Axial view. b. Sagittal view.

Given the clinical picture of non-progressive hypotonia, upper motor neuron signs, and developmental delay, coupled with no sentinel event during delivery that might explain this presentation, the child underwent further investigation to find the underlying cause. A 1.5 Tesla Brain Magnetic Resonance Imaging (MRI) was performed at two years under general anesthesia. It only showed marginal brain volume reduction with prominent cerebellar folia suggestive of cerebellar atrophy (Figure 1). 

The family was then referred to the clinical geneticist, who ordered a microarray, which did not yield a clinically significant result. Following this, whole-exome sequencing (WES-Trio) was performed on the child and the parents at the age of 30 months. It showed a novel homozygous mutation in the *AARS2* gene (specifically in chr6_44270253) for which both parents were heterozygous carriers (Table 1). At that time, this mutation was classified as a variant of uncertain significance (VUS) given that the phenotype of the patient did not entirely fit the previously reported scarce clinical cases of *AARS2* mutation and because the mutation was an intronic variant, flanking the splice site acceptor sequence of exon 18. The report also suggested close observation of this mutation, as functional analysis revealed that it has a functional impact on splicing by in silico tools. After this mutation was discovered, the child underwent ophthalmological screening, an echocardiography, and an electrocardiography for cardiac evaluation, all of which returned as normal.

**Table 1:** Result of whole exome sequencing done for the patient and showing the homozygous AARS2 mutation Confirmed by Sanger sequencing as a homozygous mutation in both directions in the index. Confirmed by Sanger sequencing as a heterozygous mutation in both directions in both parents. AARS2- Alanyl-TRNA Synthetase 2; COX8-Combined oxidative phosphorylation deficiency-8; LKENP: Leukoencephalopathy, Progressive, With Ovarian Failure; AR – Autosomal Recessive; Homo – Homozygous; VUS – Variant of uncertain clinical significance; MOI – Mode of Inheritance; NA- Not Applicable; rsID- Reference SNP cluster ID; SIFT - Sorting Intolerant From Tolerant.

Summary of diagnostic variants detected										
Gene	Associated Disease	Chromosome	DNA Change	Protein Change	rsID	MOI	Zygosity	Effect on Protein (Polyphen)	Effect on Protein (SIFT)	Type
AARS2	COXPD8/LKENP	chr6_44270253 G>C	NA	Splice site region	Novel	AR	Homozygous	NA	NA	VUS

During the clinical workup, the child was referred to rehabilitation services and was subsequently started on intensive physiotherapy, occupational therapy, and hydrotherapy. As per the parents at that time, this has helped the child initially with her tone and power, and she began vocalizing a few sounds. She remained under follow-up with pediatric neurology. At the age of eight years, she started to develop spasticity in both her upper and lower limbs, so she was started on baclofen, with weight-adjusted and response-adjusted doses, to some clinical benefit. At the age of nine years, she started to develop dystonic posturing in her upper and lower limbs, so she was started on clonidine with marginal improvement. Currently at 10 years of age, she remains globally delayed and is dependent on her parents and caretakers for all her activities of daily living. Revisiting the presently published literature, it’s now believed that the mutation is likely pathogenic in nature, supported by the evolving clinical phenotype and the elevated lactate from the child's blood sample.

## Discussion

**Figure 2:**
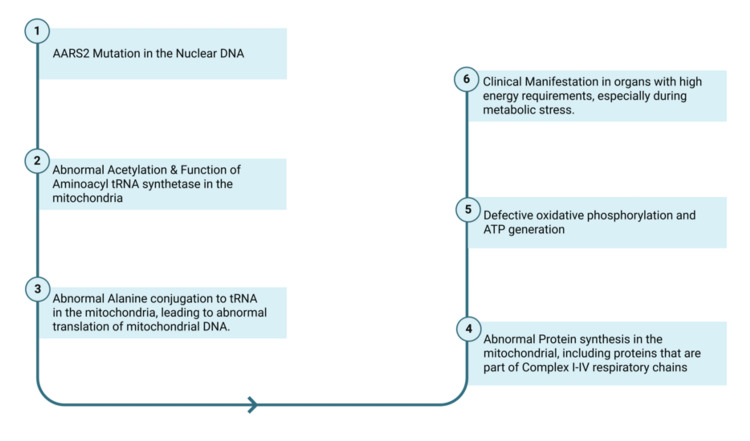
Suggested pathogenesis of AARS2 mutation AARS2: Alanyl-tRNA Synthetase 2 Created with BioRender.com.

Alanyl-tRNA Synthetase 2 (*AARS2*) is a gene located on chromosome 6 and is responsible for aminoacylation of tRNA in the mitochondria, an essential step in mRNA translation inside the mitochondria [5]. This is necessary for protein synthesis, which is subsequently used for respiratory complex synthesis inside the mitochondria. As mitochondria are responsible for oxidative phosphorylation, dysfunction explains the clinical findings of patients with these mutations, as it impairs the function of organs with high ATP requirements like the heart and the brain [2]. The proposed pathogenesis is explained in the diagram below (Figure 2).

**Figure 3:**
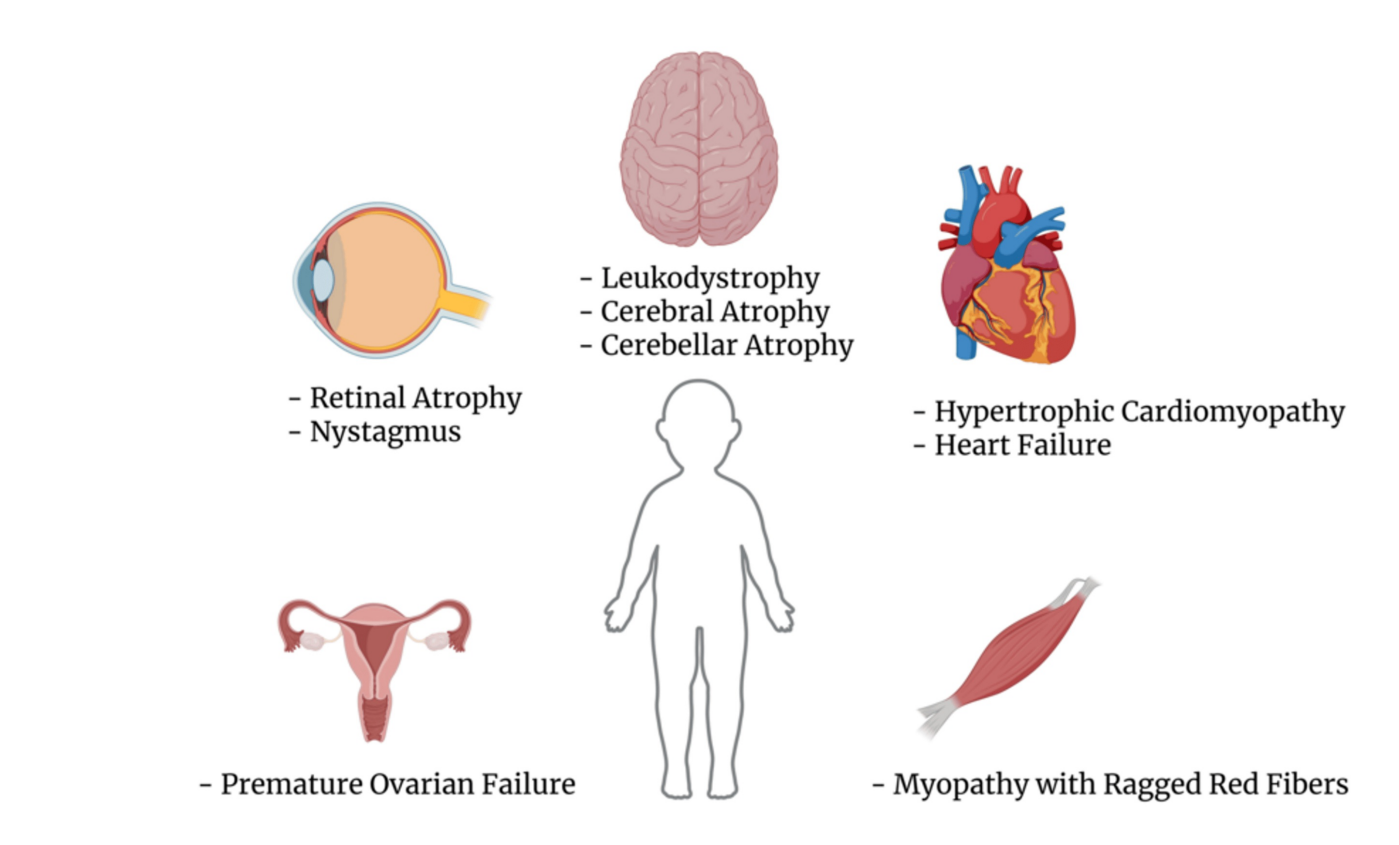
Clinical spectrum of AARS2 mutation AARS2: Alanyl-tRNA Synthetase 2 Created with BioRender.com.

The known clinical phenotype associated with this condition has been expanded with the identification of further mutations in the gene and individual cases with various clinical manifestations. From tremor, nystagmus, and primary amenorrhea without leukoencephalopathy in adults [6] to retinopathy and optic atrophy in children [7] are just a few of the myriad signs and symptoms. In the first 10 years after the mutation was discovered, the clinical phenotype was mainly either lethal infantile hypertrophic cardiomyopathy caused by combined oxidative phosphorylation deficiency or progressive adult-onset Leukoencephalopathy with ovarian failure (LKENP), but this has now expanded further [8,9] (Figure 3). Euro et al. (2015) set the groundwork for how the different mutations in this gene will lead to different clinical manifestations [1]. They did this via the discovery that the gene is responsible for three different segments of the enzyme (an editing portion for correction of improper amino acetylation, an acetylating portion and a C terminal portion) and different mutations in different parts of the gene responsible for different parts will lead to different clinical manifestations [1,5].

As suggested by Heath et al. (2025), the definition of mitochondrial disease must evolve from strictly describing generalized clinical syndromes and phenotypes (like LKENP in AARS2 mutations) and start describing mitochondrial disorders based on the specific gene that is mutated and causing the phenotype. This will help direct research and develop precision medicine for treating these diseases [10]. The authors suggest referring to the widening phenotype of the *AARS2* mutation as an entity of (*AARS2*-mutation related disease), instead of calling it by the separate clinical syndromes that this mutation causes.

## Conclusions

*AARS2* gene mutations result in a mitochondrial disease with variable clinical manifestations dependent on the exact mutation. However, it is still related to organs with high energy requirements (the heart, the brain, and the eyes). To the best of the authors’ knowledge, this is the first case of a novel AARS2 mutation described in a 17-month-old child from the Middle East region, with earlier onset and varying progressive neurological symptoms compared to previous reported cases.
